# First record of the family Colinauropodidae (Myriapoda, Pauropoda) in China, with the description of three new species

**DOI:** 10.3897/zookeys.947.53723

**Published:** 2020-07-08

**Authors:** Yun Bu

**Affiliations:** 1 Natural History Research Center, Shanghai Natural History Museum, Shanghai Science & Technology Museum, Shanghai 200041, China Shanghai Science & Technology Museum Shanghai China

**Keywords:** anal plate, bothriotricha, pauropod, sclerotized plate, taxonomy

## Abstract

The pauropod family Colinauropodidae Scheller, 1985 is recorded from China for the first time. Three new species of the genus *Colinauropus* Remy, 1956 are described: *Colinauropus chinensis***sp. nov.** and *C. chongzhoui***sp. nov.** from Jiangsu Province, and *C. foliosus***sp. nov.** from Sichuan Province. They can be easily separated from similar species by the number and the shape of sclerotized plates on the tergites, setae on the body and the anal plate. A key for all species of the genus is provided.

## Introduction

The family Colinauropodidae Scheller, 1985 includes the single genus *Colinauropus* Remy, 1956 and contains three species in the world: *Colinauropus regis* Remy, 1956 from Réunion and Mauritius (Remy 1956, 1959), *C. schelleri* Hagino, 1991 from Japan (Hagino 1991, 2005), and *C. haginoi* Scheller, 2009 from Philippines (Scheller, 2009). Their most charming character lies in the tergites which split into several distinctly sclerotized plates of irregular shape (Scheller 2011).

In the original description, the genus *Colinauropus* was considered to be affiliated with species of the family Brachypauropodidae Silvestri, 1902 according to the fragmented tergites and the shape of anal plate (Remy 1956). Its taxonomic position was reconsidered and placed in the family Pauropodidae Lubbock 1867, under the new subfamily Colinauropodinae, which was supposed to be closely related to the subfamily Scleropauropodinae (Scheller 1985). In the latest classification system, the subfamily Colinauropodinae was upgraded to family Colinauropodidae (Scheller 2009, 2011).

The purposes of this study are 1) to record the occurrence of family Colinauropodidae Scheller, 1985 in China for the first time; 2) to describe three new species of the genus *Colinauropus* Remy, 1956 from China; 3) to give a key to the species of the genus.

## Materials and methods

All pauropods were collected using a Tullgren’s funnel. The specimens were sorted under a stereomicroscope and preserved in 80% alcohol. They were mounted on slides using Hoyer’s solution and dried in an oven at 50 °C. Observations were performed under a phase contrast microscope (Leica DM 2500). Photos were taken using a digital camera (Leica DMC 4500). Line drawings were made using a drawing tube. All specimens were deposited in the collection maintained by the Shanghai Natural History Museum.

Abbreviations used in the descriptions follow Qian et al. (2018). Absolute lengths of all other body parts are given in mm and μm. Otherwise, the text refers to relative lengths. For the description of the new species, measurements and indices of paratypes are given in brackets.

## Results

### Taxonomy

#### Family Colinauropodidae Scheller, 1985

##### Genus Colinauropus

Taxon classificationAnimalia

Remy, 1956

191295C3-BC2B-5306-8ED0-67D15A1596B1

###### Type species.

*Colinauropus regis* Remy, 1956.

###### Diagnosis.

Body fusiform; head and pygidium free; tergites divided into sclerotized coarse plates, partly of irregular shape; stalk of antennal globulus *g* shorter than globulus itself; adults with first and last pair of legs 5-segmented, remaining pairs 6-segmented; pygidial sternum with two pairs of setae *b*_1_+*b*_2_ (Scheller 2011).

###### Distribution.

Ethiopian, Palaearctic, and Oriental regions.

##### Colinauropus chinensis sp. nov.

Taxon classificationAnimalia

5A7DA4AC-BB77-5F41-85CC-275419CF4B79

http://zoobank.org/DFA53888-8023-4745-B84C-82E51BCB5E57

Figures 1 , 2 , 3

###### Material examined.

***Holotype***, female adult with 9 pairs of legs (slide no. JS-WX-PA2017033), China, Jiangsu Province, Wuxi City, Daji Mountain, extracted from soil samples in bamboo forest, elev. 5 m, 31°32'N, 120°12'E, 9-X-2017, coll. Y. Bu. ***Paratypes***, 2 female adults with 9 pairs of legs (slides no. JS-WX-PA2017031, JS-WX-PA2017032), same data as holotype; 1 female adult with 9 pairs of legs (slide no. JS-WX-PA2018006), same locality as holotype, 9-X-2018, coll. Y. Bu.

###### Diagnosis.

*Colinauropus chinensis* sp. nov. is characterized by the cylindrical, annulate setae on head, antennae and tergites; tergite I without distinct sclerotized plates; tergite II with 2 large and 4 small sclerotized plates; tergites III–V each with 4 large and 4 small plates; tergite VI with 2 large plates; seta *st* on tergum of pygidium cylindrical; bothriotrichum *T*_3_ with thicker axis and dense tufted pubescence distally.

###### Description.

Adult body length (0.88–) 0.96 (–0.98) mm (*N* = 4); body white-yellow in alcohol, sclerotized plates on tergites brown (Fig. 2A).

**Figure 1.**
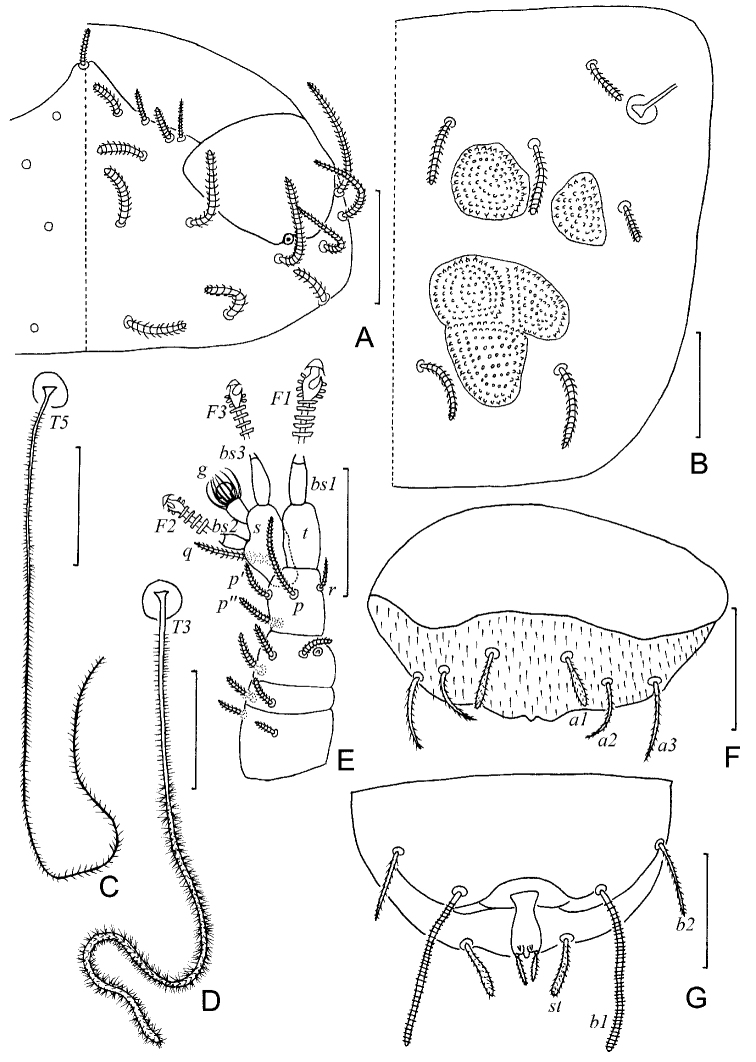
*Colinauropus chinensis* sp. nov. **A** head, dorsal view, right side **B** tergite II, right side **C***T*_5_**D***T*_3_**E** right antenna, tergal view **F** tergum of pygidium **G** sternum of pygidium and anal plate. Scale bars: 20 μm.

**Figure 2.**
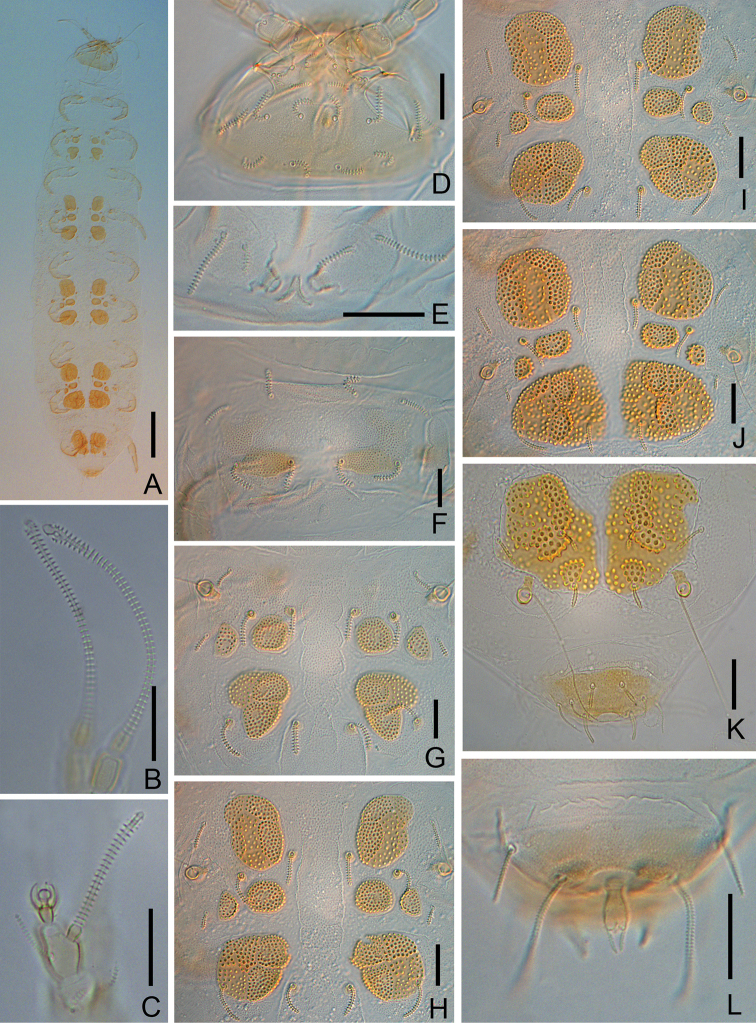
*Colinauropus chinensis* sp. nov. **A** habitus, tergal view **B***F*_1_ and *F*_3_ of right antenna **C** globulus and *F*_2_ of right antenna **D** head, dorsal view **E** collum segment, sternal view **F** tergite I **G** tergite II **H** tergite III **I** tergite IV **J** tergite V **K** tergite VI and tergum of pygidium **L** sternum of pygidium and anal plate. Scale bars: 100 μm (**A**); 20 μm (**B–L**).

***Head*** (Figs 1A, 2D). Dorsal setae cylindrical, annulate, first and second rows shorter than posterior rows. Relative lengths of setae, 1^st^ row: *a*_1_ = 10, *a*_2_ = 8 (–9); 2^nd^ row: *a*_1_ = 13 (–14), *a*_2_ = 7 (–9), *a*_3_ = 7; 3^rd^ row: *a*_1_ = (18–) 20, *a*_2_ = (23–) 24; 4^th^ row: *a*_1_ = 16 (–17), *a*_2_ = 16 (–17), *a*_3_ = 22 (–25), *a*_4_ = 14 (–16); lateral group setae *l*_1_ =21 (–26), *l*_2_ = 26 (–31), *l*_3_ = 29 (–35); the ratio *a*_1_/*a*_1_–*a*_1_ in 1^st^ row 0.7 (–0.9), 2^nd^ row 0.5, 3^rd^ row1.2 and 4^th^ row 0.7 (–0.8). Temporal organs oval in dorsal view, their length 0.8 of their shortest distance apart. Pistil present. Head cuticle faintly granular.

***Antennae*** (Figs 1E, 2B, C). Antennal segments 1–3 with 2, 2, 3 short, cylindrical, annulate setae respectively, and 1 rudimentary setae present on segment 3. Antennal segment 4 with 4 cylindrical setae; relative lengths of setae: *p* = 10, *p*’ = 6, *p*’’ = 5, *r* = 5; tergal seta *p* (1.3–) 1.4 times as long as tergal branch *t*; the latter cylindrical, 1.7 (–1.8) times as long as its greatest diameter and 0.8 of sternal branch *s*, which itself is 1.6 times as long as its greatest diameter. Seta *q* cylindrical, annulate, 0.9 of *s.* Relative lengths of flagella (base segments included) and base segments: *F*_1_ = 100, *bs*_1_ = 8 (–11); *F*_2_= (41–) 49, *bs*_2_ = (5–) 6; *F*_3_ = (84–) 92, *bs*_3_ = 9 (–10). *F*_1_ (6.6–) 7.2 times as long as *t*, *F*_2_ and *F*_3_ (2.3–) 2.7 and (4.8–) 5.1 times as long as *s* respectively. Distal calyces spherical; apex of flagella fusiform, with a short lateral flap. Globulus *g* 1.7 times as long as wide; about 12 bracts, capsule spherical; width of *g* (0.5–) 0.6 of the greatest diameter of *t.* Antennal cuticle granulated.

***Trunk*.** Setae on collum segment cylindrical, annulate; sublateral setae length (20–) 22 μm, (1.9–) 2.0 times as long as submedian setae; sternite process triangular, furcate and granulated; appendages barrel shaped (Fig. 2E). Tergite I with 4+4 short, cylindrical setae (14–15 μm), posteriorly with two patches of thickened cuticles but not form distinct sclerotized plates (Fig. 2F); Tergite II with 6+6 setae (9–20 μm), 4 small anterior and 2 large posterior sclerotized plates (Figs 1B, 2G); Tergites III–V each with 6+6 setae (9–21 μm), 4 large and 4 small sclerotized plates (Fig. 2H–J); Tergite VI with 4+2 setae and 2 large plates (Fig. 2K), posterior setae 10 (–11) μm long, their mutual distance 20 (–23) μm (Fig. 2K). Sclerotized plates with dense, brown granules, diameter 1.5–3.2 μm (Fig. 1B). Other areas of cuticle on tergites with pale and fine granules.

***Bothriotricha*.** Relative lengths: *T*_1_ = 100, *T*_2_ = (110–) 117, *T*_3_ = (122–) 128, *T*_4_ =133(–140), *T*_5_ = (167–) 178. *T*_1_, *T*_2_, *T*_4_ and *T*_5_ long, with short erect and oblique pubescence on axis (Fig. 1C). *T*_3_ with thicker axis and dense tufted pubescence distally (Fig. 1D).

**Figure 3.**
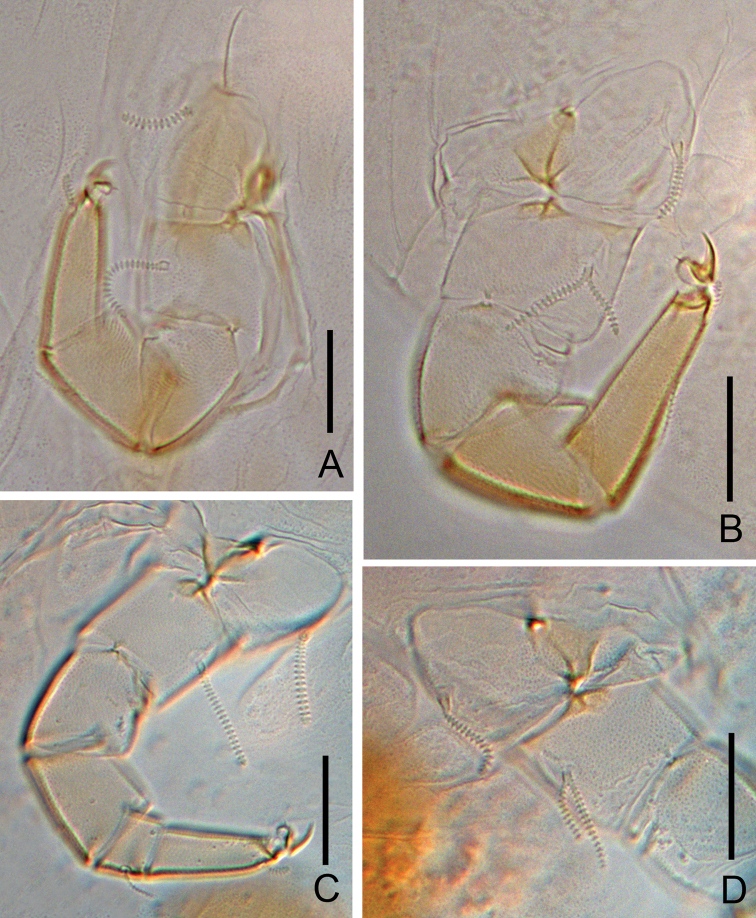
*Colinauropus chinensis* sp. nov. **A** leg 1 **B** leg 9 **C** leg 4 **D** coxa and trochanter of leg 9. Scale bars: 20 μm.

***Legs*.** First and last pair of legs 5-segmented, others 6-segmented (Fig. 3A–C). Setae on coxa and trochanter of legs 1–8 cylindrical, annulate (Fig. 3A, C), length 13 (–14) μm and 18 (–20) μm respectively. Setae on coxa of leg 9 cylindrical, annulate, length (15–) 17 μm (Fig. 3B, D). Setae on trochanter of leg 9 furcate, with subcylindrical, annulate, blunt branches, shorter one about (0.6–) 0.7 of longer one (Fig. 3B, D). Tarsi 1–8 with short, annulate distal seta (6 μm) only (Fig. 3A, C). Tarsus of leg 9 tapering, 35 μm in length, 3.2 (–3.5) times as long as its greatest diameter (Fig. 3B), proximal seta slender, pointed, striate, 10 (–13) μm in length; distal seta cylindrical, annulate, 6 (–7) μm in length, about 0.2 of the tarsal length. Cuticle of tarsus pubescent.

***Pygidium*. *Tergum***. Posterior margin waved. Relative lengths of setae: *a*_1_ = 10, *a*_2_ = 13, *a*_3_ = 15, *st* = 10 (–12). Setae distinctly differentiated, *a*_1_ short, clavate, pubescent; *a*_2_ and *a*_3_ slender and pubescent (Figs 1F, 2K); *st* thick and pubescent (Figs 1G, 2K). Distance *a*_1_–*a*_1_ as same long as *a*_1_; distance *a*_1_–*a*_2_ 2.0 (–2.5) times as long as *a*_2_–*a*_3_; distance *st*–*st* (1.5–) 1.6 times as long as *st* and 1.6 (–1.8) times as long as distance *a*_1_–*a*_1_.

***Sternum*** (Figs 1G, 2L). Posterior margin with a deep indention between *b*_1_. Relative lengths of setae (*a*_1_ =10): *b*_1_ = 33(–35), *b*_2_ =13 (–15). Seta *b*_1_ cylindrical, thick and annulate; *b*_2_ slender and short, pubescent. Distance *b*_1_–*b*_1_ (0.7–) 0.8 of length of *b*_1_; distance *b*_1_–*b*_2_ (0.7–) 0.9 of *b*_2_.

***Anal plate*** linguiform, glabrous, 2.0 times longer than broad, lateral margins concave in anterior part, posterior margin with three small lobes; two pairs of appendages present: inner one tiny and conical; outer one cylindrical and longer, (0.4–) 0.5 of the length of plate and with short pubescence (Figs 1G, 2L).

###### Etymology.

The species is named after China where the type specimens were collected.

###### Distribution.

China (Jiangsu). Only known from the type locality.

###### Remarks.

*Colinauropus chinensis* sp. nov. is most similar to *C. haginoi* Scheller, 2009 from Philippines in the similar shape of the anal plate and absence of sclerotized plates on tergite I. They can be easily distinguished by the number of sclerotized plates on tergites II and VI (6 and 2 in *C. chinensis* sp. nov., vs. 8 and 4 in *C. haginoi*), length of setae on collum segment (sublateral setae 1.9–2.0 times as long as submedian setae in *C. chinensis* sp. nov. vs. 3.2 times in *C. haginoi*), and the shape of *T*_3_ (subcylindrical, not clavate in *C. chinensis* sp. nov. vs. proximal half distinctly clavate in *C. haginoi*).

##### Colinauropus chongzhoui sp. nov.

Taxon classificationAnimalia

0A12C370-33C2-59FE-BF57-9E431DAF2B1E

http://zoobank.org/9DB912C3-DB79-4AC1-8649-AAAC2985E274

Figures 4 , 5 , 6

###### Material examined.

***Holotype***, female adult with 9 pairs of legs (slide no. JS-WX-PA2018007), China, Jiangsu Province, Wuxi City, Daji Mountain, extracted from soil samples in bamboo forest, elev. 5 m, 31°32'N, 120°12'E, 8-X-2018, coll. Y. Bu. Non-type specimens, 1 juvenile with 8 pairs of legs (slides no. JS-WX-PA2017034), 2 juveniles with 6 pairs of legs (slides no. JS-WX-PA2018008, JS-WX-PA2018009), same data as holotype.

###### Diagnosis.

*Colinauropus chongzhoui* sp. nov. is characterized by the slender, annulate-striate setae on head, antennae and tergites; tergite I with 1 large sclerotized plate; tergite II with 6 small and 2 large sclerotized plates; tergites III–V each with 4 large and 4 small plates; tergite VI with 2 large plates; seta *st* on tergum of pygidium clavate; bothriotrichum *T*_3_ brush-shaped, with branched pubescence distally.

###### Description.

Adult body length 0.97 mm (*N* = 1); body white-yellow in alcohol, sclerotized plates on tergites brown (Fig. 5A).

**Figure 4.**
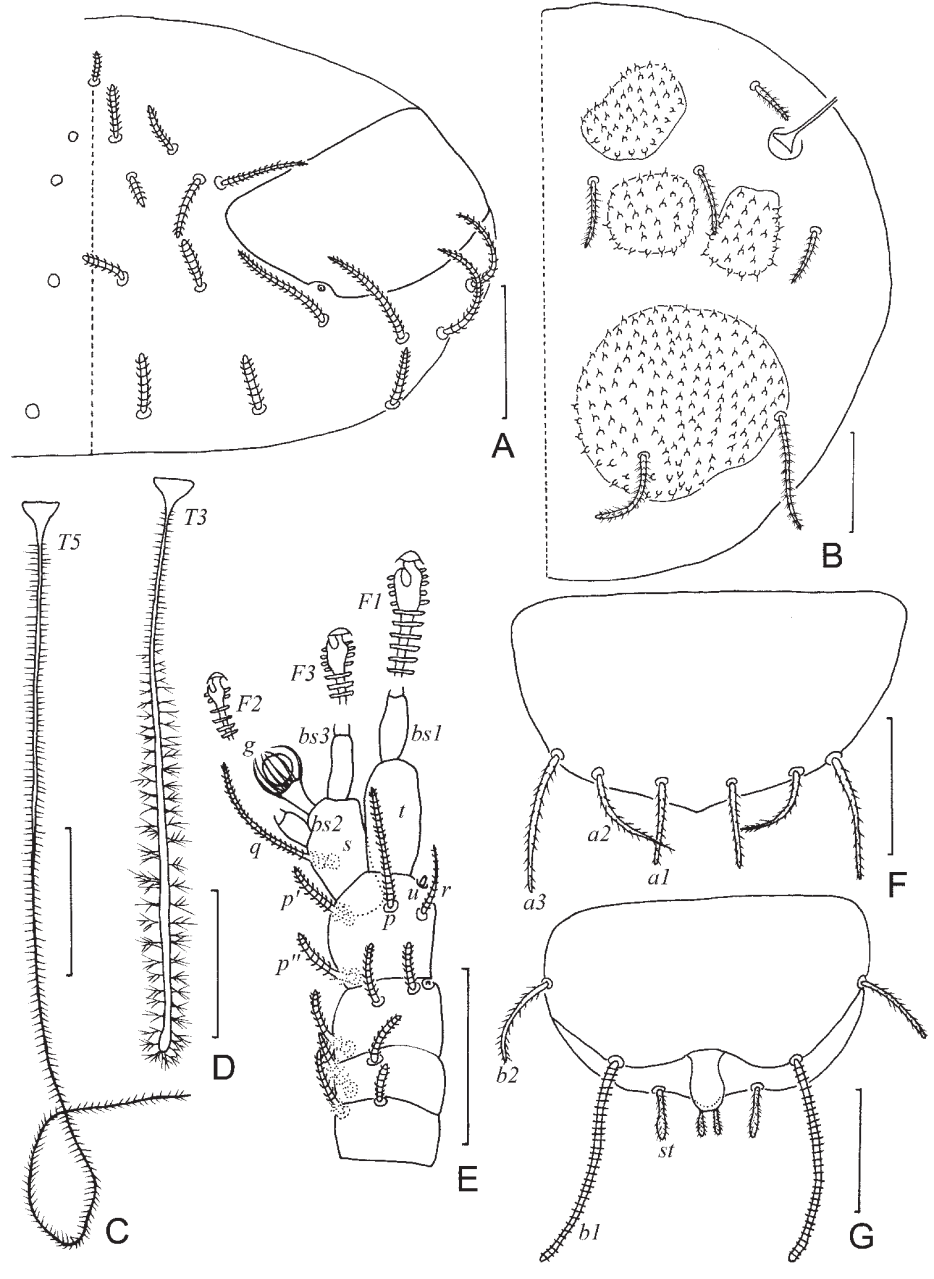
*Colinauropus chongzhoui* sp. nov. **A** head, dorsal view, right side **B** tergite II, right side **C***T*_5_**D***T*_3_**E** right antenna, tergal view **F** tergum of pygidium **G** sternum of pygidium and anal plate. Scale bars: 20 μm.

**Figure 5.**
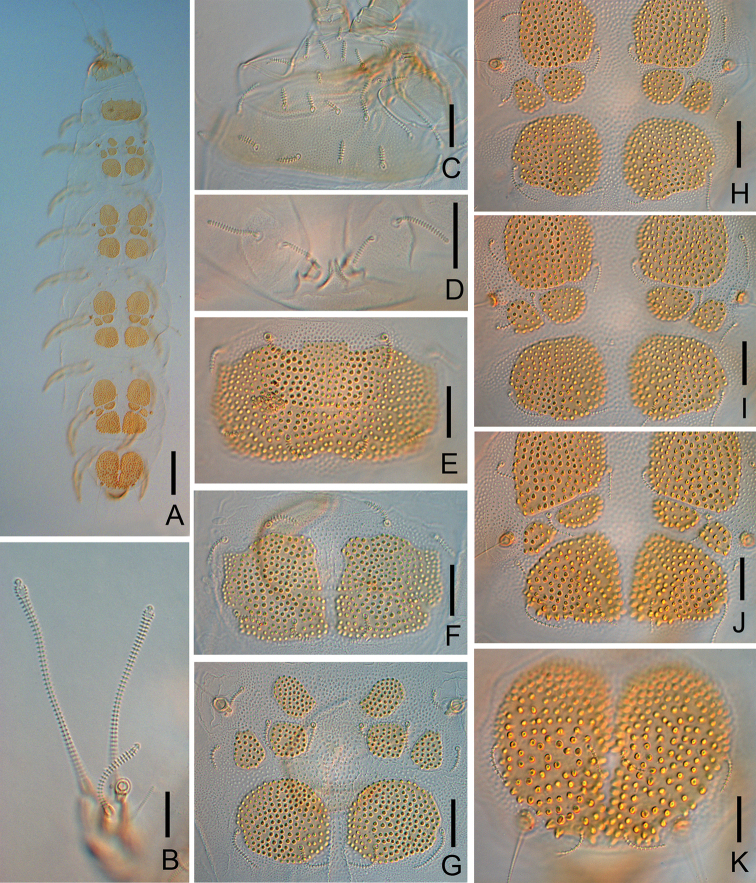
*Colinauropus chongzhoui* sp. nov. **A** habitus, tergal view **B** left antenna, sternal view **C** head, dorsal view **D** collum segment, sternal view **E** tergite I of adult **F** tergite I of juvenile **G** tergite II **H** tergite III **I** tergite IV **J** tergite V **K** tergite VI. Scale bars: 100 μm (**A**); 20 μm (**B–K**).

***Head*** (Figs 4A, 5C). Dorsal setae short, cylindrical, annulate-striate, except seta *a*_3_ of second row which is slender and tapering. Relative lengths of setae, 1^st^ row: *a*_1_ = 10, *a*_2_ = 10; 2^nd^ row: *a*_1_ = 8, *a*_2_ = 14, *a*_3_ = 14; 3^rd^ row: *a*_1_ = 9, *a*_2_ =10; 4^th^ row: *a*_1_ = 12, *a*_2_ = 14, *a*_3_ = 21, *a*_4_ = 12; lateral group setae *l*_1_ =23, *l*_2_ = 21, *l*_3_ = 19; the ratio *a*_1_/*a*_1_–*a*_1_ in 1^st^ row 1.5, 2^nd^ row 0.6, 3^rd^ row 0.9 and 4^th^ row 0.7. Temporal organs oval in dorsal view, their length 1.1 times as long as their shortest distance apart. Pistil present. Head cuticle with dense granules.

***Antennae*** (Figs 4E, 5B). Antennal segments 1–3 with 2, 2, 3 short, cylindrical, annulate setae respectively, and 1 rudimentary seta present on segment 3. Antennal segment 4 with 4 cylindrical setae and rudimentary seta *u*; relative lengths of setae: *p* = 10, *p*’ = 6, *p*’’ = 6, *r* = 6, *u* = 1; tergal seta *p* 1.1 times as long as tergal branch *t*; the latter cylindrical, 2.2 times as long as its greatest diameter and 0.9 of sternal branch *s*, which itself is 1.9 times as long as its greatest diameter. Seta *q* cylindrical, annulate, 1.3 times as long as *s.* Relative lengths of flagella (base segments included) and base segments: *F*_1_ = 100, *bs*_1_ = 10; *F*_2_ = 52, *bs*_2_ = 5; *F*_3_ = 88, *bs*_3_ = 9. *F*_1_ 6.4 times as long as *t*, *F*_2_ and *F*_3_ 2.9 and 4.9 times as long as *s* respectively. Distal calyces spherical; apex of flagella fusiform, with a short lateral flap. Globulus *g* 1.7 times as long as wide; about 12 bracts, capsule spherical; width of *g* 0.5 of the greatest diameter of *t.* Antennal cuticle densely granulated.

***Trunk*.** Setae on collum segment cylindrical, annulate; sublateral setae length 23 μm, 2.1 times as long as submedian setae; sternite process triangular, furcate and granulated; appendages cylindrical and tapering (Fig. 5D). Tergite I with 4+4 cylindrical setae (12–13 μm) and 1 large sclerotized plate (Fig. 5E) (2 plates in juveniles, Fig. 5F); Tergite II with 6+6 setae (12–23 μm), 6 small anterior and 2 large posterior sclerotized plates (Figs 4B, 5G); Tergites III–V each with 6+6 setae (6–27 μm), 4 large and 4 small sclerotized plates (Fig. 5H–J); Tergite VI with 4+2 setae and 2 large plates (Fig. 5K), posterior setae 23 μm long, their mutual distance 18 μm (Fig. 5K). Sclerotized plates with dense, brown granules, diameter 2–4 μm, and each granule with one short straight apical hair (Fig. 4B). Other areas of cuticle on tergites with coarse granules.

**Figure 6.**
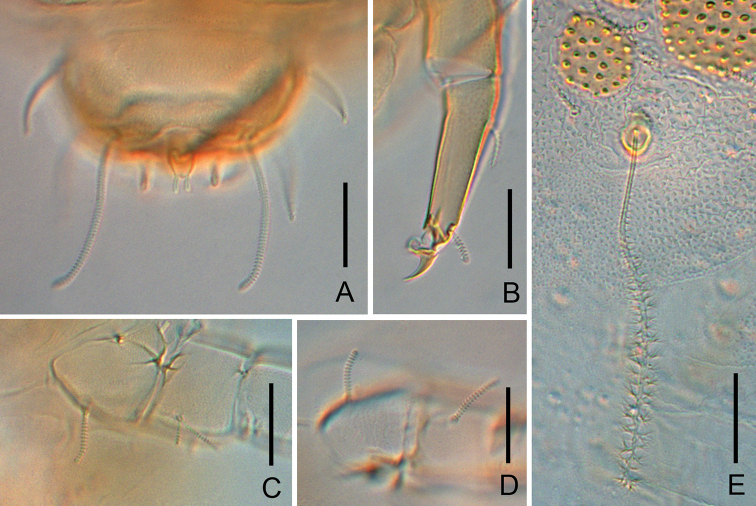
*Colinauropus chongzhoui* sp. nov. **A** sternum of pygidium and anal plate **B** tarsus of leg 9 **C** coxa and trochanter of leg 9 **D** coxa and trochanter of leg 1 **E***T*_3_. Scale bars: 20 μm.

***Bothriotricha*.** Relative lengths: *T*_1_ = 100, *T*_2_ = 113, *T*_3_ = 86, *T*_4_ =118, *T*_5_ = 167. *T*_1_, *T*_2_, *T*_4_ and *T*_5_ thin, long, with short erect or oblique pubescence on axis (Fig. 4C). *T*_3_ brush-shaped, with thicker axis and branched pubescence in distal 2/3 (Figs 4D, 6E).

***Legs*.** First and last pair of legs 5-segmented, others 6-segmented. Setae on coxa and trochanter of legs 1–8 cylindrical, annulate, length 13–15 μm and 16–20 μm respectively (Fig. 6D). Setae on coxa of leg 9 cylindrical, annulate, length 12 μm (Fig. 6C). Seta on trochanter of leg 9 furcate, with two subcylindrical, annulate, blunt branches, shorter one about 0.5 of longer one (Fig. 6C). Tarsi 1–8 with short annulate distal seta (8 μm) only. Tarsus of leg 9 tapering, 40 μm in length, 3.6 times as long as its greatest diameter (Fig. 6B), proximal seta slender, pointed, striate, 11 μm in length; distal one cylindrical, annulate, 9 μm in length, about 0.2 of the tarsal length. Cuticle of tarsus pubescent.

***Pygidium*. *Tergum***. Posterior margin blunt triangular. Relative lengths of setae: *a*_1_ = 10, *a*_2_ = 12, *a*_3_ = 16, *st* = 5. Setae distinctly differentiated, *a*_1_ cylindrical, pubescent; *a*_2_ and *a*_3_ slender, pubescent (Fig. 4F); *st* short, clavate, pubescent (Figs 4G, 6A). Distance *a*_1_–*a*_1_ 0.7 of length of *a*_1_; distance *a*_1_–*a*_2_ 1.6 times as long as *a*_2_–*a*_3_; distance *st*–*st* 2.0 times as long as *st* and 1.5 times as long as distance *a*_1_–*a*_1_.

***Sternum*** (Figs 4G, 6A). Posterior margin with one lower indention between *b*_1_. Relative lengths of setae (*a*_1_ =10): *b*_1_ = 28, *b*_2_ =12. Seta *b*_1_ cylindrical, thick, annulate; *b*_2_ slender, short, pubescent. Distance *b*_1_–*b*_1_ 0.8 of length of *b*_1_; distance *b*_1_–*b*_2_ 0.9 of *b*_2_.

***Anal plate*** linguiform, 1.7 times longer than broad; a pair of clavate appendage inserted posteriorly, 0.4 of the length of plate, and with short pubescence (Figs 4G, 6A).

###### Etymology.

The species is dedicated to the honor of the late Professor Chongzhou Zhang (1930–2014) who was an eminent zoologist from Institute of Zoology, Chinese Academy of Sciences, for his great contribution to the knowledge of Myriapoda of China (Stoev et al. 2014).

###### Distribution.

China (Jiangsu). Only known from the type locality.

###### Remarks.

*Colinauropus chongzhoui* sp. nov. is similar to *Colinauropus regis* Remy, 1956 in the shape of anal plate. They can be easily distinguished by the number of sclerotized plates on tergite I (1 large plate in *C. chongzhoui* sp. nov. vs. 2 in *C. regis*) and tergite II (8 in *C. chongzhoui* sp. nov., vs. 6 in *C. regis*), shape of setae on tergites (slender and striate in *C. chongzhoui* sp. nov. vs. clavate and pubescent in *C. regis*), and the shape of seta *a*_1_ on pygidium (tapering in *C. chongzhoui* sp. nov. vs. clavate in *C. regis*).

##### Colinauropus foliosus sp. nov.

Taxon classificationAnimalia

812FD209-31A5-56D6-B74B-EBC507B4ED45

http://zoobank.org/867E5192-CE51-4339-947B-7C17D668C86E

Figures 7 , 8 , 9

###### Material examined.

***Holotype***, female adult with 9 pairs of legs (slide no. SC-PA2017002), China, Sichuan Province, Ganzi Tibetan Autonomous Region, Kangding City, Yala town, 30°06'N, 101°57'E, elev. 3100 m, soil samples from mixed forest, 11-VIII-2017, coll. C.W. Huang. ***Paratypes***, 1 male adult with 9 pairs of legs (slides no. SC-PA2017001) and 1 female adult with 9 pairs of legs (slide no. SC-PA2017003), same data as holotype.

###### Diagnosis.

*Colinauropus foliosus* sp. nov. is characterized by the leaf-shaped pubescent setae on head and tergites; tergite I with one large sclerotized plate; tergites II–IV each with 4 large and 4 small plates; tergite V with 4 large and 2 small middle sclerotized plates; tergite VI with 2 large plates; granules on plates ovoid, each inserted with one fine hair; seta *st* on tergum of pygidium clavate; bothriotrichum *T*_3_ with thick axis and dense tufted pubescence distally.

###### Description.

Adult body length 1.28 (–1.32) mm (*N* = 3); body white-yellow in alcohol, sclerotized plates on tergites brown (Fig. 8A).

**Figure 7.**
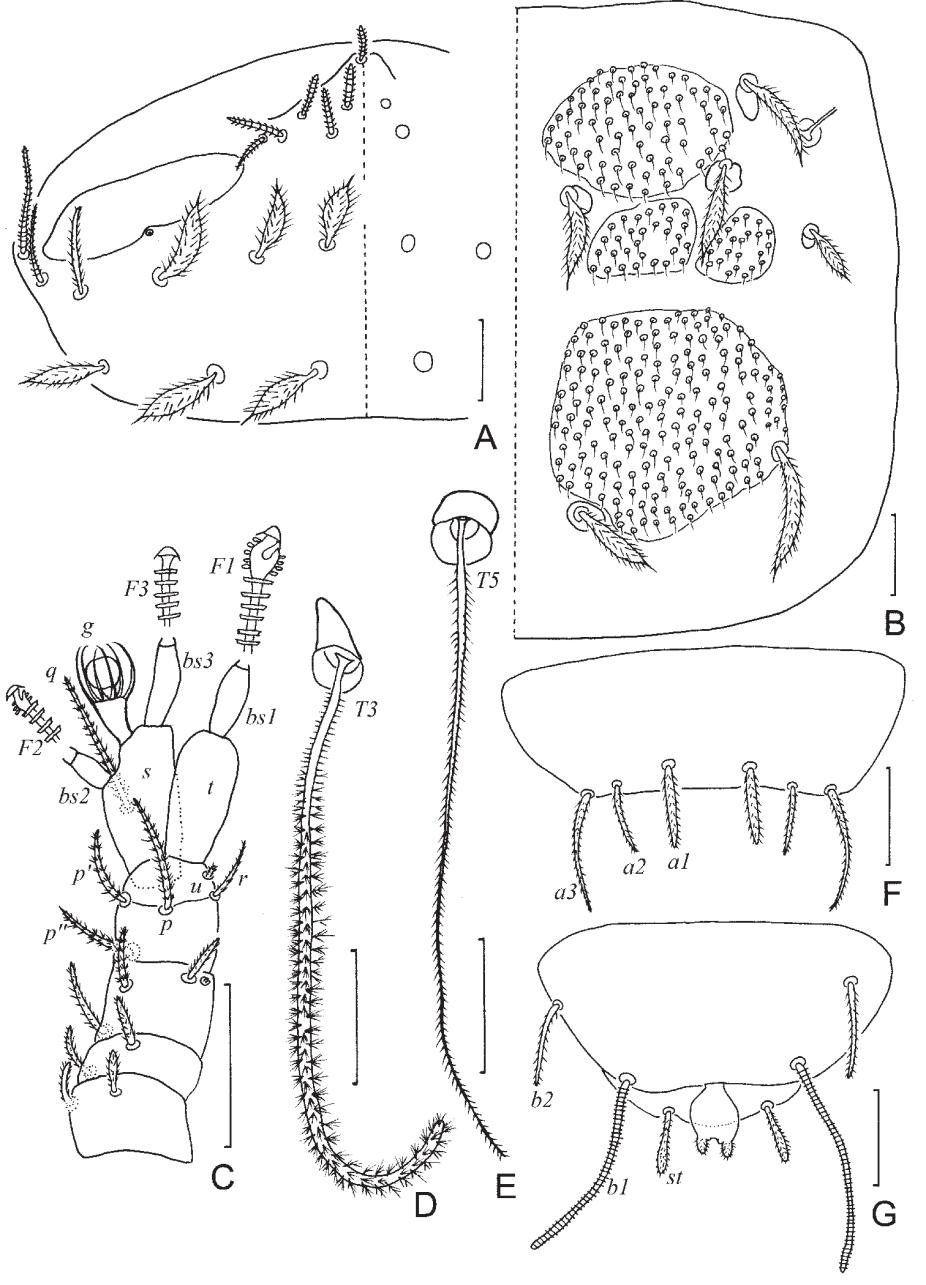
*Colinauropus foliosus* sp. nov. **A** head, dorsal view, left side **B** tergite II, right side **C** right antenna, tergal view **D***T*_3_**E***T*_5_**F** tergum of pygidium **G** sternum of pygidium and anal plate. Scale bars: 20 μm.

**Figure 8.**
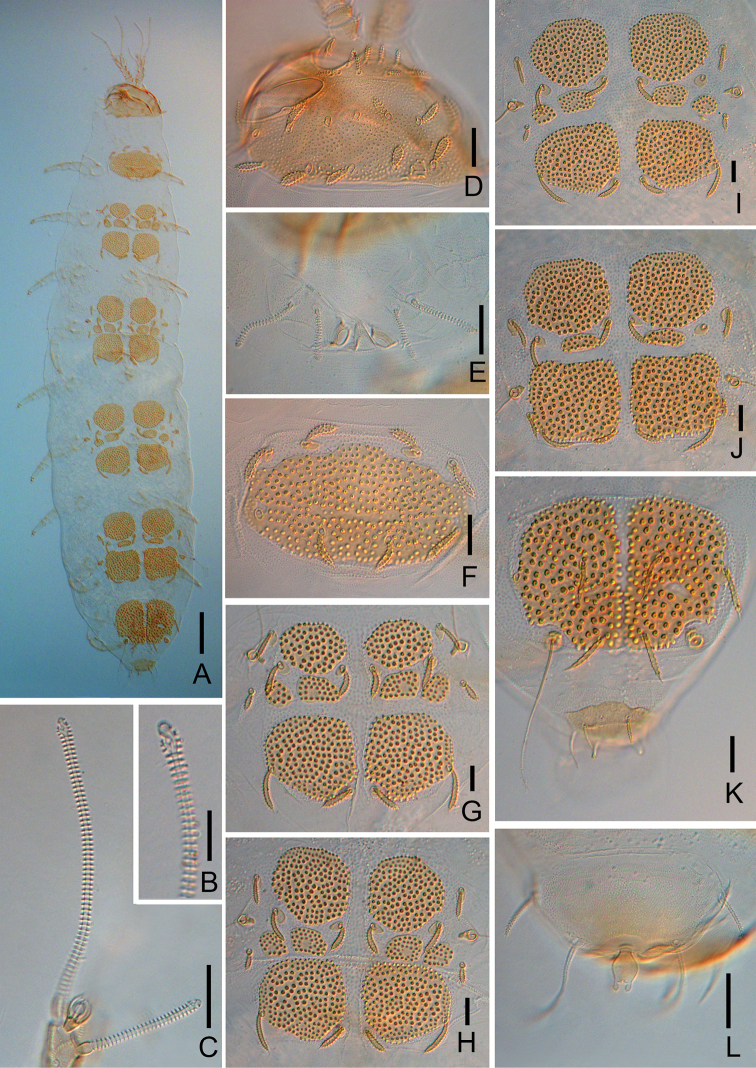
*Colinauropus foliosus* sp. nov. **A** habitus, tergal view **B** terminal part of *F*_1_**C** sternal branch of left antenna, show *F*_2_, *F*_3_ and globulus **D** head, dorsal view **E** collum segment, sternal view **F** tergite I **G** tergite II **H** tergite III **I** tergite IV **J** tergite V **K** tergite VI and tergum of pygidium **L** sternum of pygidium and anal plate. Scale bars: 100 μm (**A**); 20 μm (**B–K**).

***Head*** (Figs 7A, 8D). Dorsal setae distinctly differentiated, on first and second rows cylindrical to tapering; on third and fourth rows leaf-shaped and with long pubescence; seta *a*_3_ of second row slender and tapering. Relative lengths of setae, 1^st^ row: *a*_1_ = 10, *a*_2_ = 10 (–12); 2^nd^ row: *a*_1_ = 10 (–11), *a*_2_ = (11–) 12, *a*_3_ = 12 (–13); 3^rd^ row: *a*_1_ = (18–) 20, *a*_2_ = 18 (–20); 4^th^ row: *a*_1_ = 13 (–16), *a*_2_ = (15–) 17, *a*_3_ = 20 (–23), *a*_4_ = 16 (–17); lateral group setae *l*_1_ =18 (–24), *l*_2_ = 18 (–23), *l*_3_ = 25(–32); the ratio *a*_1_/*a*_1_–*a*_1_ in 1^st^ row (1.6–) 1.7, 2^nd^ row (0.7–) 0.8, 3^rd^ row1.0 (–1.1) and 4^th^ row 0.7 (–0.8). Temporal organs oval in dorsal view, their length (0.8–) 0.9 of their shortest distance apart. Pistil present. Head cuticle with coarse granules.

***Antennae*** (Figs 7C, 8B, C). Antennal segments 1–3 with 2, 2, 3 short cylindrical pubescent setae respectively, and 1 rudimentary seta present on segment 3. Antennal segment 4 with 4 tapering setae and a short, rudimentary *u*; relative lengths of setae: *p* = 10, *p*’ = 7 (–8), *p*’’ = (6–) 7, *r* = 5 (–6), *u* = 1; tergal seta *p* (0.9 of –) 1.0 times as long as tergal branch *t*; the latter cylindrical, 1.8 (–2.0) times as long as its greatest diameter and 0.7 (–0.9) of sternal branch *s*, which itself about 2.0 times as long as its greatest diameter. Seta *q* cylindrical, annulate, 0.8 (–1.0 times as long as) of *s.* Relative lengths of flagella (base segments included) and base segments: *F*_1_ = 100, *bs*_1_ = 8 (–11); *F*_2_= (35–) 42, *bs*_2_ = (4–) 5; *F*_3_ = (78–) 93, *bs*_3_ = 7 (–9). *F*_1_ (5.8–) 8.6 times as long as *t*, *F*_2_ and *F*_3_ 2.0 (–2.1) and 4.4 (–4.8) times as long as *s* respectively. Distal calyces spherical; apex of flagella fusiform, on *F*_1_ and *F*_3_ with a short lateral flap. Globulus *g* 1.7 times as long as wide; about 12 bracts, capsule spherical; width of *g* (0.4–) 0.6 of the greatest diameter of *t.* Antennal cuticle granulated.

**Figure 9.**
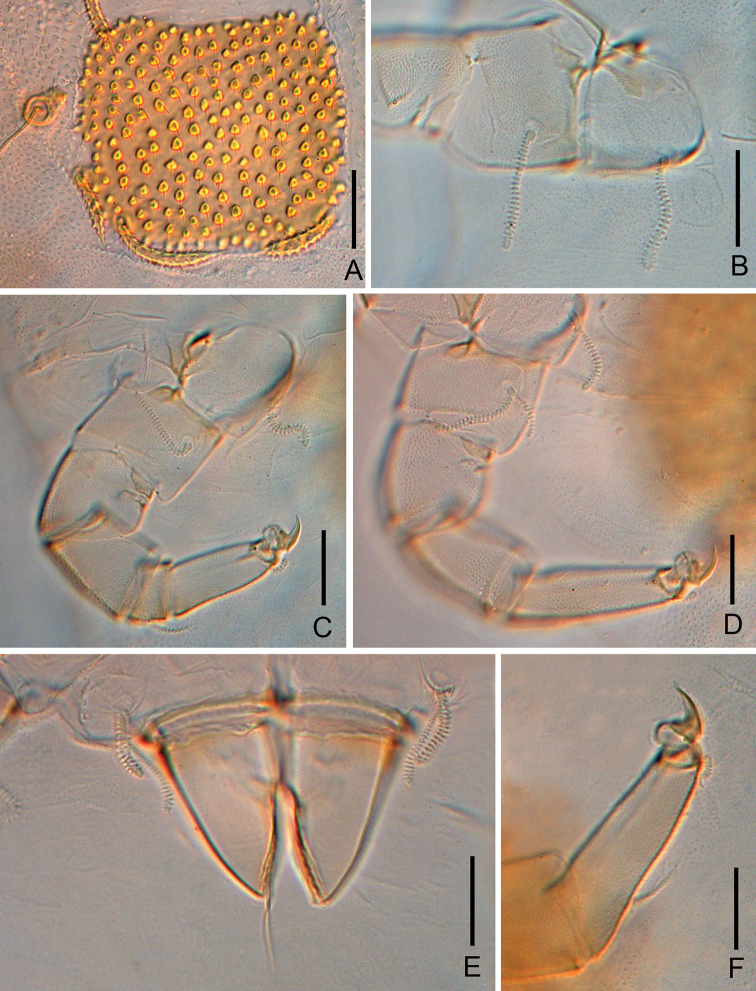
*Colinauropus foliosus* sp. nov. **A** posterior plate on tergite V, left side **B** coxa and trochanter of leg 1 **C** leg 8 **D** leg 9 **E** male genital papillae and coxa of leg 2 **F** tarsus of leg 9. Scale bars: 20 μm.

***Trunk*.** Setae on collum segment cylindrical, annulate; sublateral setae length 22 (–34) μm, (1.4–) 1.7 times as long as submedian setae; sternite process triangular, furcate and granulated; appendages tapering (Fig. 8E). Tergite I with 4+4 leaf-shaped setae (22–27 μm) and 1 large sclerotized plate (Fig. 8F); Tergites II–IV each with 6+6 setae (18–40 μm), 4 large and 4 small sclerotized plates (Figs 7B, 8G–I); Tergite V with 6+6 slender setae (20–40 μm), 4 large and 2 small sclerotized plates, posterior plates square (Figs 8J, 9A); Tergite VI with 4+2 setae and 2 large plates (Fig. 8K), posterior setae 35 μm long, their mutual distance 24 (–26) μm (Fig. 8K). Sockets of some setae on tergites and bothriotricha with distinct thickened cuticle surrounded (Figs 7B, D, E, 8F–J). Sclerotized plates with ovoid, brown granules, diameter 1.5–5.0 μm and each with one long curved hair (Figs 7B, 9A). Cuticle granulated or pubescent.

***Male genital papillae*** (Fig. 9E) glabrous, subuliform, 1.5 times as long as greatest diameter; seta 0.5 of the length of papilla. Seta on coxa of leg 2 in male with two adjacent setae (only 1 thick setae in female, 20–25 μm), both cylindrical and annulate, one thick and short, 17 μm in length, another slender and longer, 20 μm in length (Fig. 9E).

***Bothriotricha*.** Relative lengths: *T*_1_ = 100, *T*_2_ = (110–) 100, *T*_3_ = (95–) 105, *T*_4_ =114 (–120), *T*_5_ = (115–) 120. *T*_1_, *T*_2_, *T*_4_ and *T*_5_ thin, long, with short erect pubescence on axes (Fig. 7E). *T*_3_ cylindrical, with thicker axis and dense tufted pubescence in distal 2/3 part (Fig. 7D).

***Legs*.** First and last pair of legs 5-segmented, others 6-segmented (Fig. 9C, D). Setae on coxa and trochanter of legs 1–8 cylindrical, annulate (Fig. 9B), length 23 (–26) μm and 23 (–27) μm respectively. Seta on coxa of leg 9 cylindrical, annulate, length 22 (–25) μm (Fig. 9D). Seta on trochanter of leg 9 furcate, with subcylindrical, annulate, blunt branches, shorter branch about 0.5 of longer one (Fig. 9D). Tarsi 1–8 with short, annulate distal seta (6–8 μm) only (Fig. 9C). Tarsus of leg 9 tapering, 48 (–55) μm in length, 3.7 (–4.2) times as long as its greatest diameter (Fig. 9F), proximal seta slender, pointed, pubescent, 12 (–15) μm in length; distal one cylindrical, annulate, 6 (–8) μm in length, about 0.1 of the tarsal length. Cuticle of tarsus pubescent.

***Pygidium*. *Tergum***. Posterior margin straight. Relative lengths of setae: *a*_1_ = 10, *a*_2_ = (8–) 9, *a*_3_ = (12–) 16, *st* = (7–) 9. Setae *a*_1_ cylindrical, pubescent; *a*_2_ and *a*_3_ slender and pubescent (Figs 7F, 8K); *st* clavate, pubescent (Fig. 7G). Distance *a*_1_–*a*_1_ (0.6–) 0.8 of *a*_1_; distance *a*_1_–*a*_2_ 2.0 (–2.5) times as long as *a*_2_–*a*_3_; distance *st*–*st* (1.5–) 1.6 times as long as *st* and (1.7–) 2.0 times as long as distance *a*_1_–*a*_1_.

***Sternum*** (Figs 7G, 8L). Posterior margin straight between *b*_1_. Relative lengths of setae (*a*_1_ =10): *b*_1_ = (25–) 29, *b*_2_ =14 (–15). Seta *b*_1_ cylindrical, thick, annulate; *b*_2_ tapering, short, pubescent. Distance *b*_1_–*b*_1_ (0.7–) 0.8 of length of *b*_1_; distance *b*_1_–*b*_2_ (0.6–) 0.7 of *b*_2_.

***Anal plate*** round, glabrous, 1.5 times longer than broad, lateral margins bulged in middle part, posterior part divided into two round, pubescent branches, two tiny lobes present at inner side (Figs 7G, 8L).

###### Etymology.

The species name “*foliosus*” from the Latin “foliose”, leaf-shaped, referring to the leaf-shaped setae on head and tergites.

###### Distribution.

China (Sichuan). Only known from the type locality.

###### Remarks.

*Colinauropus foliosus* sp. nov. differs from all other congeners by having 6 sclerotized plates on tergite V, and the posterior two square-shaped, compared with 4 or 8 irregular plates in congeners. It is similar to *C. regis* Remy, 1956 in the leaf-shaped setae on tergites, but they can be easily distinguished by the number of sclerotized plates on tergite I and II (1 and 8 in *C. foliosus* sp. nov. vs. 2 and 6 in *C. regis*), shape of the setae in the posterior two rows of the head (leaf-shaped in *C. foliosus* sp. nov. vs. cylindrical in *C. regis*), and the shape of anal plate (with two round posterior branches in *C. foliosus* sp. nov. vs. with two clavate appendages in *C. regis*).

### Key to the species of the genus *Colinauropus* Remy, 1956

**Table:** 

1	Tergite I without sclerotized plates, at most with two posterior thickened patches	**2**
–	Tergite I with distinct sclerotized plates	**3**
2	Tergite II with 4 large and 4 small sclerotized plates, tergite VI with 4 plates	***C. haginoi* Scheller, 2009 (Philippines)**
–	Tergite II with 2 large and 4 small sclerotized plates, tergite VI with 2 plates	***C. chinensis* sp. nov. (China)**
3	Tergite I with 1 large sclerotized plate	**4**
–	Tergite I with 2 sclerotized plates	**5**
4	Setae on head and tergite cylindrical, tergite II with 2 large and 6 small sclerotized plates	***C. chongzhoui* sp. nov. (China)**
–	Setae on head and tergite leaf-shaped, tergite II with 4 large and 4 small sclerotized plates	***C. foliosus* sp. nov. (China)**
5	Tergites II and V with 6 and 8 sclerotized plates respectively, anal plate rounded with 2 posterior appendages, setae on tergites II–V clavate	***C. regis* Remy, 1956 (Réunion, Mauritius)**
–	Tergites II and V with 8 and 4 sclerotized plates respectively, anal plate indented mediodistally without appendages, setae on tergites II–V cylindrical	***C. schelleri* Hagino, 1991 (Japan)**

## Discussion

The genus *Colinauropus* Remy, 1956 is well defined by the presence of sclerotized plates on the tergites. The number of plates on tergites I, II, V and VI, which varies from 1 to 8, are good characters for species identification, while tergites III and IV always have 8 plates in all species. The shape and arrangement of the plates are also taxonomically informative for species definition. On tergite I, the plates are absent or at most with small patches of thickened cuticle posteriorly (*C. haginoi*, *C. chinensis* sp. nov.), with 1 complete large plate (*C. chongzhoui* sp. nov., *C. foliosus* sp. nov.) or with 2 axially separated plates (*C. regis*, *C. schelleri*). On tergite II, 4 small anterior plus 2 large posterior plates are present in *C. regis* and *C. chinensis* sp. nov., 6 small anterior plus 2 large posterior plates are present in *C. chongzhoui* sp. nov., while there are 4 small plus 4 large plates in the remaining three species. On tergite V, the number of plates can be 4 in *C. schelleri*, 4 large plus 2 small middle plates in *C. foliosus* sp. nov., and 4 large plus 4 small plates in others. On tergite VI, all species have 2 large plates, except *C. haginoi* which has 4 plates. The shapes of plates are usually ovoid, round, sub-triangular, or irregular, while the two posterior large plates on tergite V are nearly square-shaped in *C. foliosus* sp. nov. Variation of plates within a species has never been reported in former studies but is observed here in *C. chongzhoui* sp. nov., which exhibits 2 plates on tergite I in juveniles vs. 1 complete plate in adults. Thus, caution is advised when describing species of this genus, which should be based on fully mature specimens. As an additional taxonomic character, the bothriotrichum *T*_3_ is also well differentiated and nicely separates species. The most informative characters are the shape and appendages of the anal plates. The six known species of the genus *Colinauropus* Remy, 1956 can be distinguished by the key provided above.

## Supplementary Material

XML Treatment for Colinauropus

XML Treatment for Colinauropus chinensis

XML Treatment for Colinauropus chongzhoui

XML Treatment for Colinauropus foliosus
